# Hunting field: insights on distribution pattern of bacteria and immune cells in solid tumors

**DOI:** 10.1093/nsr/nwab023

**Published:** 2021-02-06

**Authors:** Yang Li, Yuqi Wang, Xuefei Li, Chenli Liu

**Affiliations:** CAS Key Laboratory for Quantitative Engineering Biology, Shenzhen Institute of Synthetic Biology, Shenzhen Institutes of Advanced Technology, Chinese Academy of Sciences, China; University of Chinese Academy of Sciences, China; CAS Key Laboratory for Quantitative Engineering Biology, Shenzhen Institute of Synthetic Biology, Shenzhen Institutes of Advanced Technology, Chinese Academy of Sciences, China; CAS Key Laboratory for Quantitative Engineering Biology, Shenzhen Institute of Synthetic Biology, Shenzhen Institutes of Advanced Technology, Chinese Academy of Sciences, China; University of Chinese Academy of Sciences, China; CAS Key Laboratory for Quantitative Engineering Biology, Shenzhen Institute of Synthetic Biology, Shenzhen Institutes of Advanced Technology, Chinese Academy of Sciences, China; University of Chinese Academy of Sciences, China

Bacterial cancer therapy, which was first applied in the clinic in 1868, has regained attention owing to the recent progress made in synthetic biology. Considering their easily manipulated genomes, preferential accumulation in tumors, and penetration abilities, bacteria have shown great therapeutic potential in tumor treatment. During treatment, it has been found that bacteria in tumors lead to corresponding changes in the abundance as well as locations of a variety of cells and substances, especially immune cells, forming a unique distribution pattern. This has been suggested to contribute to the therapeutic effect of bacterial cancer therapy [1].

**Figure 1.**
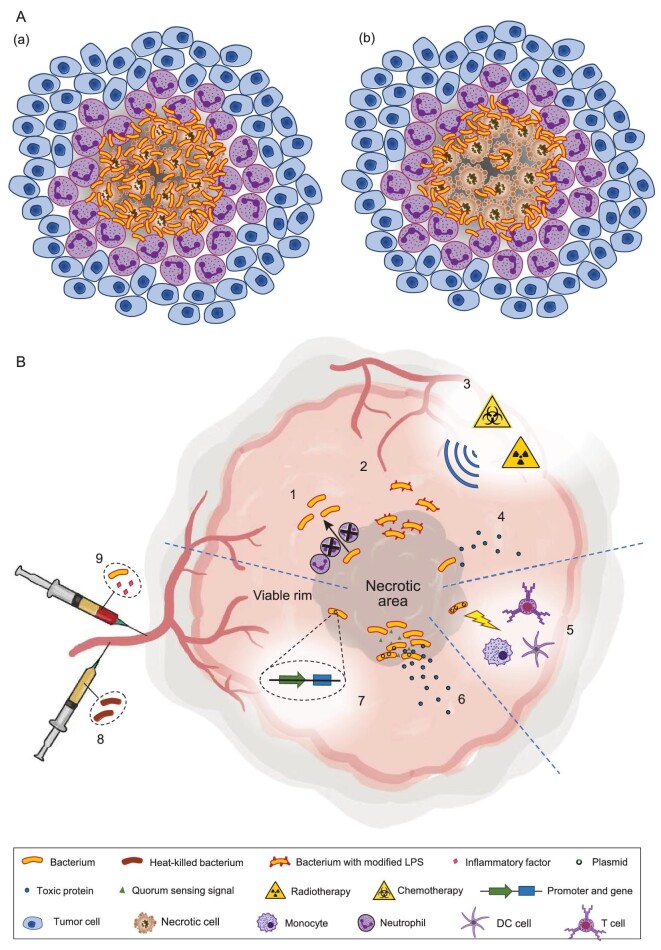
(A) The distribution pattern of bacteria in a tumor. Bacteria mainly colonize the necrotic/hypoxia area of a tumor, which can be divided into two modes: (a) evenly distributing throughout the necrotic area or (b) accumulating in the hypoxic area with a few colonies deeper in the necrotic area. Neutrophils gather around the necrotic area and form a ring to surround the bacteria, which sometimes overlaps with part of the bacterial area. (B) Proposals for optimizing bacterial therapy. The methods can be divided into three aspects. One is to target the viable area in the tumor by preventing the formation of the neutrophils ring (1), modifying the bacteria to have the potential of escaping the confinement of the neutrophil ring (2), combining bacteria with chemotherapy and radiotherapy (3) or designing bacteria to secrete drug proteins which could spread to the viable rim (4). Another is to introduce plasmids expressing tumor antigens/cytokines/immunostimulators/immunosuppressive/checkpoint inhibitor or other proteins with immunomodulatory activities into bacteria to enhance the bacterial stimulation on the immune system (5). The third is to reduce the side effects elicited by the bacteria and ensure safety by controlling the synthesis and secretion of toxic proteins or immuno-regulatory factors specifically within the tumor tissue by quorum-sensing system (6) or tumor-specific promoter (7), pre-exposing mice to heat-killed bacteria (8) or co-injecting the attenuated bacteria with inflammatory factors (9).

Generally, one to three days after the administration of *Salmonella, Clostridium, Escherichia* or *Pseudomonas* in mouse models, a relatively stable distribution pattern of bacteria and immune cells can be observed in tumors [2–4]. The stable distribution pattern (Fig. 1A) shares a common feature: bacteria mainly colonize the necrotic region of the tumor, with neutrophils forming a ring-like structure surrounding the area of bacteria. Two modes of bacterial distribution are observed: an even distribution throughout the necrotic area (Fig. 1Aa), or accumulation in the hypoxic area in close proximity to the necrotic region, with a few colonies deeper in the necrotic area (Fig. 1Ab) [2,3,5–9].

The development of the intra-tumoral distribution pattern is a dynamic process with interactions among bacteria, tumor cells and the immune system. After transport into tumor tissues through the blood stream, bacteria colonize tumors, while simultaneously the concentrations of immune factors increase as well. This results in the formation and expansion of necrotic areas in tumors, while a rim of viable tumor cells is left on the periphery. Tumors grow and create an immune-privileged environment that protects cancer cells from being easily found and killed by immune cells. After the entry of bacteria, however, the ‘peace’ is broken. Like a hunting process, bacteria are the ‘rabbits’ running into the flush forest where they can hide well, and the innate immune cells are the ‘dogs’ chasing behind. When entering the forest, rabbits and dogs wake up the sleeping ‘tigers’ (adaptive immune cells) and the other dogs. Then they find that there are also many ‘sheep’ (cancer cells) hiding there, and more tigers may come into the forest. Therefore, tigers and dogs start to hunt both the sheep and the rabbits, forming a busy and crowded ‘hunting field’.

In the ‘hunting field’, both dogs and tigers contribute to the death of sheep. The invasion of bacteria in a tumor can promote the infiltration of a large number of innate as well as adaptive immune cells. Importantly, the exhausted effector immune cells can be re-activated. There is a possibility that specific types of bacteria have similar antigens with cancer cells, which can help to stimulate the immune cells that can recognize neoantigens. Furthermore, the death of cancer cells caused by bacteria and innate immune cells may expose the neoantigens to the adaptive immune system, which could further enhance the cancer-specific killing. Therefore, when, where and how the bacteria interact with the immune system impacts the effectiveness of therapy.

This hunting field not only reflects the state of the tumor during treatment, but also affects the curative potential of bacterial treatment. Based on the distribution pattern, we can analyze and utilize the colonization mode of bacteria and overcome known limitations to optimize therapy. The recurrence of tumors after bacterial treatment is due to the proliferation of tumor cells within the viable tumor area, especially for large tumors [10]. This suggests taking additional measures to target the rim of viable cells (Fig. 1B1–4). Enhancing the antitumor effect of the immune system can be another potential method (Fig. 1B5), e.g. by increasing infiltration or anti-neoplastic activity of immune cells induced by bacteria. It is also crucial to balance toxicity and efficacy of bacterial therapy. Measures should be applied to reduce the side effects of bacteria to alleviate the harm on normal tissues, ensuring safety (Fig. 1B6–9).

Nevertheless, some questions require further exploration. This spatial pattern can be important for the survival of bacteria, but whether this prolonged existence of bacteria in tumors helps or limits the therapeutic effects still awaits an answer. It remains unclear why some genetically engineered bacteria show poor therapeutic effect and fail to induce a similar spatial pattern in a tumor. It is vital to know which abstracted appendages or gene products of bacteria contribute to the formation of a spatial pattern. In addition, although the immunology of the tumor microenvironment has been intensively studied, the behavior and effect of immune cells after bacteria have entered the tumor tissue are still obscure. Which immune cells or immune factors have crucial impacts on therapeutic effects remains to be elucidated.

Currently, the primary methods used in studies of intra-tumoral patterns are immunofluorescence and immunohistochemical staining of tumor sections. Therefore, only static images and snapshots of the spatiotemporal evolution can be captured. The continuous and dynamic characterization of different components in the whole tumor, before and after bacterial treatment, is lacking. We should note that even for primary tumors, the spatiotemporal evolution of the tumor microenvironment is still an active area of study. How bacteria trigger the required cancer-killing by immune cells will be an important focus of future study. To achieve this goal, standardized and quantitative data acquisition and analysis are required. Specifically, we need to quantify and understand how bacteria help to attract and activate corresponding immune cells and how the immune cells interact with cancer cells. Since the interactions among bacteria and the tumor microenvironment are complex, mathematical models can be helpful for explorations of the detailed mechanisms underlying the pattern evolution. More importantly, such exploration will be helpful for indicating the potential directions of strain modifications and possible treatment strategies. The challenges are not limited to bacteria engineering, and mechanistic studies on the spatiotemporal evolution of patterns will shed light on the rational engineering of the tumor microenvironment for a safe and effective therapy.
